# Exploring the uncharted role of cell senescence in rare diseases

**DOI:** 10.1186/s13023-025-03778-1

**Published:** 2025-09-01

**Authors:** Piera Selvaggio, Esi Taci, Alessandra Barassi, Valentina Massa, Cristina Gervasini, Elena Lesma, Clara Bernardelli, Elisabetta Di Fede

**Affiliations:** 1 Department of Health Sciences, University of Milan, Via Antonio di Rudinì 8, Milano, Italy; 2 “Aldo Ravelli” Center for Neurotechnology and Experimental Brain Therapeutics, University of Milan, Milan, Italy; 3 Laboratory of Clinical Chemistry, ASST Santi Paolo e Carlo, Milan, Italy

**Keywords:** Senescence, Senescence-associated secretory phenotype, Chromatinopaties, Epigenetics, Lung diseases, Lung microenvironment

## Abstract

**Background:**

Cellular senescence is a biological process in which the cell cycle is arrested in response to DNA damage caused by different endogenous and exogenous stimuli. In senescent cells, activation of intracellular cascade induces epigenetic, morphological and metabolic changes. Among them, senescent status is characterized by an alteration of the epigenome and the establishment of a peculiar senescence-associated secretory phenotype (SASP), which contributes to the extracellular matrix remodeling and senescence spreading. Growing interest is directed towards senescence relevance both in physiological processes and in pathological ones, including rare progeroid syndromes. However, little is known about senescence contribution to the onset and development of rare diseases in which aging traits are not manifested.

**Main body:**

Here, we review the current knowledge about senescence involvement in four rare mendelian disorders of the epigenetic machinery (i.e. chromatinopathies) and four rare lung diseases, that can be considered a paradigm for understanding how epigenome alteration and aberrant microenvironment modification in senescence process might drive disease onset and progression. First, we report the main characteristics of chromatinopathies and the relation between the chromatin-related epigenetic defects and the senescence features in Sotos syndrome, Cornelia de Lange syndrome, Rett syndrome, and Kleefstra syndromes. Thereafter, we describe the pathological alteration and senescence involvement in cystic fibrosis, idiopathic pulmonary fibrosis, pulmonary arterial hypertension and lymphangioleiomyomatosis, considering them as models of rare lung diseases in which accumulation of senescent cells and their proinflammatory SASP have a central role.

**Conclusion:**

Exploring the role of senescence in different and less common diseases might promote the understanding of the senescent process as a novel player in rare disorders, for a more comprehensive vision of their complexity and the suggestion of novel possible therapeutical targets.

## Background

Cellular senescence is a physio-pathological process often established in response to several stresses upon DNA damage to prevent the propagation of injured cells. The accumulation of senescent cells and how long they are retained in tissues determine different outcomes, as, physiologically, senescence is crucial for correct embryonic development, tissue regeneration and homeostasis, and tumor suppression [1, 2]. Nevertheless, prolonged senescence can lead to tissue dysfunction, tumorigenesis, and the development of age-related diseases and other pathological conditions [2].

**Fig. 1:**
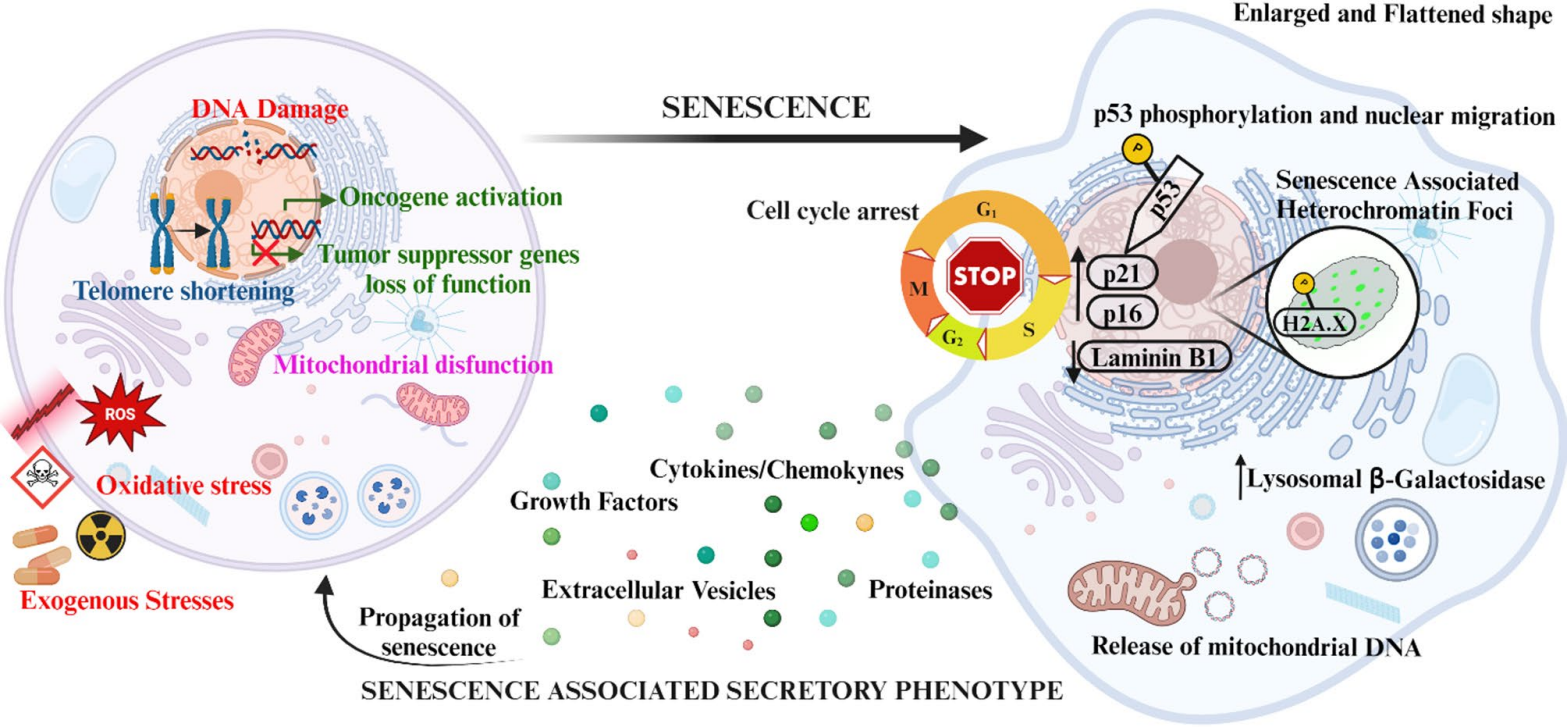
Leading stressors and biological consequences in senescence. The morphological, metabolic and molecular alterations occurring in senescent cells and relevant in the context of this review are represented: replicative senescence (in blue), stress-induced senescence (in red), oncogene-induced senescence (in green) and mitochondrial dysfunction-induced senescence (in pink) are shown on the left.

Depending on the various triggers, cellular senescence can be classified into different types. Telomere shortening and erosion induce replicative senescence [3], and several chemical agents, such as reactive oxygen species (ROS), or chemotherapeutic agents, lead to stress-induced premature senescence or SIPS [4, 5]. Moreover, the accumulation of dysfunctional mitochondria leads to a state of energy imbalance and growth arrest (mitochondrial dysfunction-induced senescence, MiDAS) [6]. Finally, the oncogene activation or the loss of function of tumor suppressor genes might induce senescence with a central role in counteracting tumor development (oncogene-induced senescence) [7] (Fig. 1, left).

Senescent cells acquire peculiar characteristics, the most common are the arrest of the cell cycle, morphological changes due to cells’ membrane and cytoskeleton modifications [8, 9], the expression of senescence-associated β-galactosidase (SA-βgal) due to the increased lysosomal biogenesis and activity [10], and the loss of nuclear integrity. Additionally, although senescent cells lose proliferative capacity, they escape the apoptotic program and remain metabolically active and secrete several specific proteins and molecules that compose the so-called Senescent-Associated Secretory Phenotype (SASP) [11] (Fig. 1, right). Hence, to assess cell senescence both in vitro and in vivo is challenging and requires the simultaneous evaluation of different markers, as, at the moment, there is not a unique hallmark that might clearly indicate the onset of this cascade.

DNA damage is the most common event that triggers senescence. After a DNA injury, such as a double-strand break, the Ataxia-Telangiectasia Mutated (ATM) protein kinase is activated primarily to induce cell cycle arrest. ATM acts by phosphorylating the tumor suppressor protein p53, which migrates into the nucleus and binds to p21^WAF/CIP1^ (p21), an inhibitor of Cyclin-Dependent Kinase (CDK), inducing its expression [9, 12]. Specifically, p21 inhibits the activity of CDK2/4 complexes, leading to cell cycle arrest between G1 and S phases.

In many cells, DNA damage and dysfunctional telomeres also induce the inhibitor of CDK p16^INK4A^ (p16) with delayed kinetics. p16 exerts its action by preventing phosphorylation of Retinoblastoma protein (Rb) operated by CDK6/4, maintaining Rb bound to the transcription factor E2F1 and thus promoting cell cycle arrest in G1/S [13]. Moreover, p16 levels are reported to increase with aging in most mammalian tissues [14]. While the p53/p21 pathway appears to play a key role in the initiation of senescence, the pathway involving p16/Rb family seems to mediate the maintenance of senescence itself [12, 13].

In addition, ATM phosphorylates Structural Maintenance of Chromosomes-1 (SMC1) and histone H2AX, which is induced by DNA damage response (DDR) activation [15, 16]. The phosphorylated form of H2A.X (γH2A.X) contributes to the development of senescence-associated heterochromatic foci, initially to silence genes involved in cell cycle progression and cell proliferation, and then spread through other loci [17]. Nuclear alteration in senescent cells also includes increased nucleoli dimension and altered integrity of nuclear lamina, at least partially due to the reduced transcription of Laminin B1 [18].

Despite growth arrest, senescent cells maintain an active metabolism and release a plethora of soluble and insoluble pro-inflammatory, pro-angiogenic and pro-proliferative molecules in the extracellular microenvironment that may alter the stromal characteristics, influencing the tissue response to different triggers and promoting physiological and pathological conditions. The SASP, or senescence-messaging secretome, comprehends the secretion of proteins, such as interleukins (ILs), chemokines (CCLs and CXCLs), growth factors and proteinases, bioactive lipids, ions, ROS and nitric oxide [11, 19]. Interestingly, multiple mechanisms of growth regulation involve SASP factors.

SASP composition depends on the cell type and is regulated at multiple levels by different nuclear and cytoplasmic factors, most of which converge in the activation of two transcription factors: NF-κB (nuclear factor of kappa light polypeptide gene enhancer in B-cells) and CEBPβ (CCAAT/enhancer binding protein β) [20]. Notably, secretion is exacerbated by p21 activation [21], while p16 is not involved in this process [22]. In addition, the mechanistic target of rapamycin (mTOR), a key regulator of cell cycle progression and cellular metabolism, induces the secretion of specific NF-κB-induced SASP factors [23]. Of note, the MiDAS stimulates a distinct SASP that lacks the canonical NF-κB/IL-1-mediated signaling but contains a different plethora of pro-inflammatory molecules [6].

SASP also includes the release of extracellular vesicles (EVs), a heterogeneous cohort of membranous particles whose composition and content (cargo) alters the behavior of target cells [24]. In senescence, p53 activation stimulates a high release of EVs with peculiar proteins, lipids and miRNAs cargo, which are different from healthy cell-derived EVs [25, 26]. All SASP factors have autocrine and paracrine activity and are involved in extracellular matrix (ECM) remodeling, immunosurveillance and senescence propagation (bystander effect) [11, 27].

In this review, we will describe the senescence contribution to rare diseases as a new paradigm for a shift between physiological and pathological state. To date, the role of senescence has been exhaustively described only for a few of them, such as progeroid syndrome [28], dyskeratosis congenita [29], and Moyamoya cardiovascular disorder [30]. Importantly, the scientific literature is growing with reports of senescence involvement in cancer and age-related disorders, possibly in association with an augmented life expectancy in many Western countries. Aside from major age-related pathologies, other human conditions have been associated with an altered equilibrium in cell cycle control. For example, several studies have shown a correlation between Down syndrome, cellular senescence and premature aging in affected individuals and in patients-derived neural progenitor cells, exhibiting global chromatin accessibility changes similar to those observed in senescent cells showing an improvement in trisomy-induced dysfunction after senolytic treatments [31].

The pathological aspects of senescence are mainly due to the impaired turnover of senescent cells that, accumulating in the tissue, cause organ dysfunction [4]; in addition, senescent cells orchestrate tissue remodeling through SASP [11]. In this context, we decided to explore the uncharted territory covering the possible contribution of cell senescence to rare diseases, with a particular focus on two groups: chromatinopathies (Sotos syndrome, Cornelia de Lange syndrome, Rett syndrome, and Kleefstra syndrome) and lung diseases (cystic fibrosis, idiopathic pulmonary fibrosis, pulmonary arterial hypertension, lymphangioleiomyomatosis). Each of the described chromatinopathies is caused by mutations in different conserved genes of the epigenetic machinery [32] and the treatments currently under investigation aim to mostly improve the neurological phenotype acting on the enzymatic activity of the epigenetic proteins involved in these disorders [33]. For this reason, highlighting the link between epigenome alterations and senescence might suggest this mechanism as a possible target and a new unexplored perspective for pharmacological intervention in chromatinopathies. Conversely, the lung diseases reported here do not share genetic bases but are all characterized by the remodeling of the lung microenvironment due to the accumulation of cells that, through secretion, induce fibrosis, inflammation and, ultimately, the impairment of lung function. Given the pro-inflammatory nature of SASP, which encompasses the large majority of the factors responsible for the manifestations characterizing the lung diseases described here, they might indicate senescence involvement in phenotype worsening, likely suggesting innovative avenues for their treatment.

## Senescence in chromatinopathies

Epigenetics refers to DNA and chromatin modifications that affect genome accessibility and gene expression. These changes can be inherited or altered by environmental factors throughout life [34]. Chromatin is regulated by epigenetic mechanisms (e.g., DNA methylation, genomic imprinting, histone modifications) through proteins involved in DNA replication and repair, cell growth, neuronal plasticity, and cognition [35–37]. These components of the epigenetic machinery have the role of writers, erasers, readers or remodelers, which can add/remove DNA methylation or histone marks (respectively writers/erasers), allow identification and interaction of epigenetic marks and link to other components of the epigenetic machinery (readers), or regulate the accessibility of DNA sites (remodelers) [38, 39]. Interestingly, researchers have recently developed machine learning-based approaches to predict and classify the functional roles of chromatin regulatory proteins, thereby improving the accuracy of the current database CREWdb, showing that many of these regulatory proteins have dual functions and contribute to multiprotein complexes, depending on their biochemical environment [40]. These mechanisms are crucial in controlling gene expression and cell fate, as shown by their involvement in several human diseases such as cancer and genetic disorders [41].

In recent times, it has also emerged that the alteration of the epigenome contributes to the senescent cell phenotype [42]. For instance, a recent study has identified p300 as a possible main driver of cellular senescence, promoting senescence-associated gene transcription by inducing *de novo* super-enhancers [43]. This protein is a histone acetyltransferase with intrinsic lysine acetyltransferase activity acting as a writer of the epigenetic machinery and it regulates chromatin opening allowing an eased access of transcription factors to DNA, therefore promoting gene transcription [33]. The action of the resulting enhancers leads to extended regions marked by H3K27ac and H3K4me1. In addition, the histone deacetylase HDAC4, involved in controlling the levels of H3K27ac, has been found to play an important role in protecting the full activation of enhancers involved in senescence [44].

It is well-known that alterations of the epigenetic machinery lead to the development of pathologies that affect chromatin structure and function, e.g. chromatinopathies or mendelian disorders of the epigenetic machinery (MDEMs), among others. This class of rare genetic disorders represents a group of overlapping syndromes caused by pathogenetic variants in genes coding for essential actors of the epigenetic machinery [32, 33, 45]. Patients affected by chromatinopathies present peculiar phenotypic features, which can differ in severity; they show common clinical signs such as intellectual disability, growth alterations, and typical facial dysmorphisms. Causative genes of MDEMs are fundamental for organ development and functioning and are indeed evolutionary conserved [45].

**Fig. 2:**
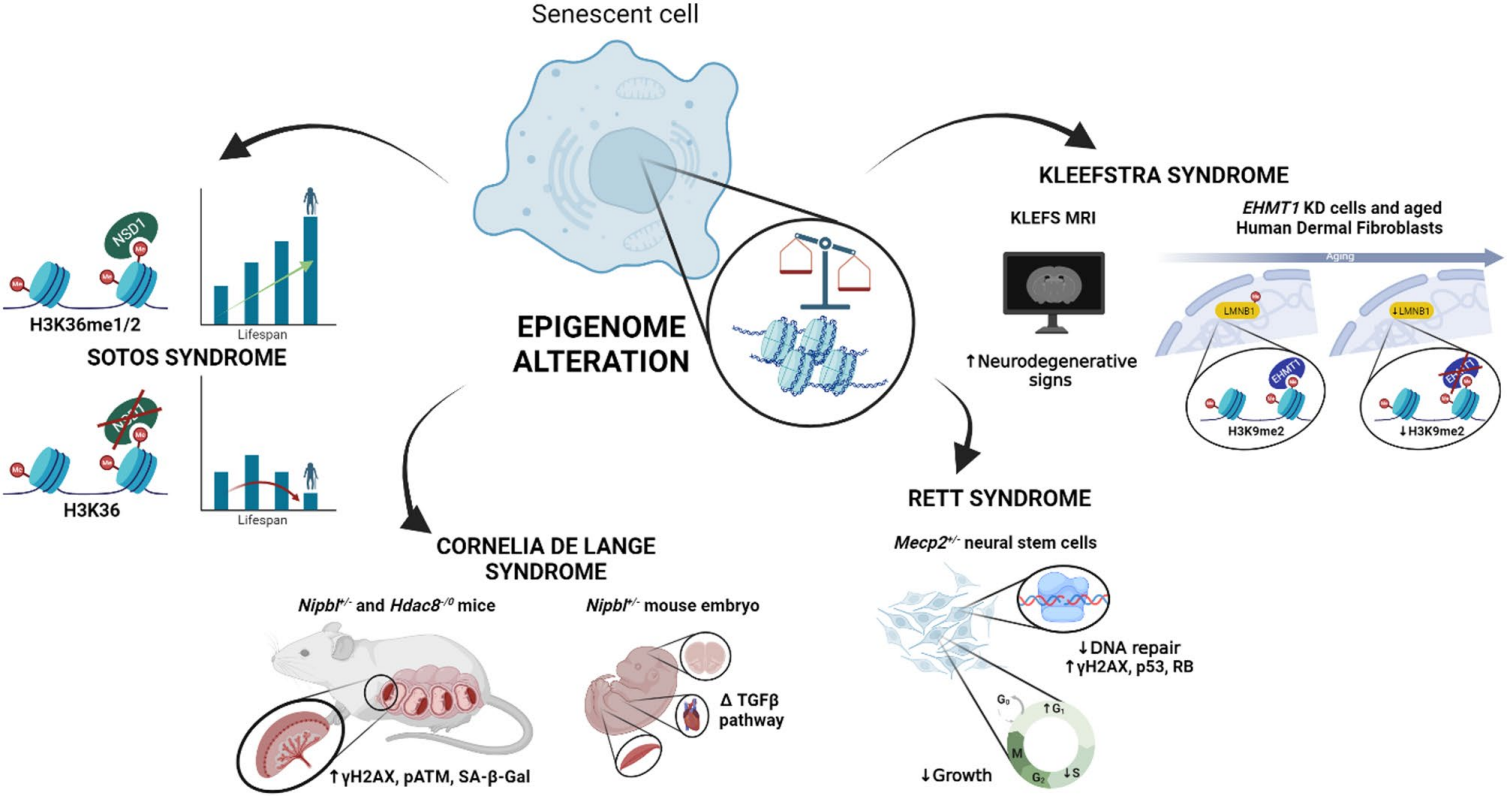
Senescence in chromatinopathies. Graphical representation of senescence features in chromatinopathies. The main markers and mechanisms of cellular senescence are highlighted.

Despite epigenetic alterations contribute both to chromatinopathies pathogenicity and to the senescence process, to date, few studies have explored the mechanism of senescence in these disorders. For this reason, here senescence features in Sotos syndrome, Cornelia de Lange syndrome, Rett syndrome, and Kleefstra syndrome are reviewed (Fig. 2).

### Sotos syndrome

Sotos syndrome (SS, OMIM #117550) is a rare multisystemic disease with an incidence of 1/10,000–14,000, which is caused in more than 95% of cases by loss-of-function heterozygous mutations in the *NSD1* gene, located at chromosome 5q35 and coding for an H3K36 methyltransferase [46], while in the minority of cases it is caused by microdeletions affecting other genes [47]. This chromatinopathy is characterized by distinctive facies, body hypergrowth in early life associated with macrocephaly, and mild to severe intellectual disability. The variety of clinical features associated with Sotos syndrome is wide.

*NSD1* mutation leads to a phenotype that includes prenatal and postnatal overgrowth, advanced bone age, developmental delay, and, in some cases, heart defects [48]. All these aging-like features allow us to consider Sotos syndrome as a potential human model of accelerated physiological aging [49, 50]. In fact, DNA methylation is considered one of the most accurate markers for biological age estimation and is the rationale for epigenetic clocks [51].

Concerning age-associated DNA methylation changes that occur between individuals, several groups have identified the so-called epigenetic clocks that accurately measure the biological/epigenetic age of an individual [52, 53]. These mathematical models were first used to predict the chronological age of an individual, however, it was observed that the epigenetic age could differ from the chronological one [54]. In addition, the epigenetic clock seems to be a conserved property in mammalian genomes [51].

Sotos causative gene encodes for a H3K36 methyltransferases playing an important role in the regulation of gene transcription [55, 56] and in DNA damage response mechanisms, among others [57]. In addition, experiments in model organisms (i.e., yeast and worm) have revealed that mutations in H3K36 methyltransferases reduce lifespan, while mutations in H3K36 demethylases increase it [58] (Fig. 2). During aging, levels of H3K36me3 decrease, leading to a loss of its ability to recruit DNA methyltransferases effectively. This leads to changes in chromatin structure and a loss of transcriptional repression in specific genomic regions, resulting in the unintended activation of normally silent areas, which can disrupt cellular functions and influence age-related gene expression changes [59].

Since it is already known that H3K27me3 levels decrease in senescent fibroblasts [60, 61], it would be highly valuable exploring H3K36, as it is poorly addressed in the current literature. Indeed, methylation of H3K36 not only plays a crucial role in aging, affecting gene regulation and lifespan, but also cellular senescence, as shown in a recent work which demonstrated that NSD1-mediated methylation of H3K36 affects the senescence state in chondrocytes [62] In addition, this epigenetic modification is part of the wider epigenetic remodeling seen in senescence, including the development of the SASP [63]. Interestingly, fibroblasts isolated from Sotos patients showed dysregulated expression of genes involved in cell cycle, proliferation, apoptosis and cellular senescence, among others, suggestive of a senescent phenotype which should be further addressed [64].

Thus, using different assessments for the epigenetic clock, it has been proved that Sotos syndrome accelerates epigenetic aging, suggesting a key role of the H3K36 methylation mechanism in epigenetic homeostasis in humans, while recent evidence points to an elucidation of senescence involvement in Sotos syndrome pathogenesis [49].

### Cornelia de Lange syndrome (CdLS)

Cornelia de Lange syndrome (CdLS) is an autosomal dominant or X-linked rare genetic disorder caused by mutations in one among at least seven different genes: *NIPBL*,* SMC1A*,* SMC3*,* RAD21*, *HDAC8*,* BRD4 and ANKRD11* (CdLS1, OMIM #122470; CdLS2, #300590; CdLS3, #610759; CdLS4, #614701; CdLS5, #300882 CdLS6, #620568; *611192). Causative variants in additional genes, such as *MAU2*, *EP300*,* AFF4 and TAF1*, can cause a CdLS-like phenotype [65, 66].

CdLS affects 1/10,000–30,000 newborns, and the diagnosis occurs during the prenatal stages or more often at birth due to distinctive features (e.g., arched eyebrows, long eyelashes, low ears, small and widely spaced teeth) and craniofacial malformations. CdLS patients show short stature, intellectual disabilities that vary among individuals, behavioral disorders (autism spectrum disorders), gastrointestinal tract disorders, microcephaly, and heart defects [67].

CdLS causative genes encode for proteins of the cohesins complex, which is fundamental in several cellular processes. Proteins belonging to the cohesin complex mediate sister chromatid cohesion during cell division, DNA replication and repair, and genome organization [68].

Several animal models for this syndrome, such as zebrafish and mice, showed alterations in specific gene expression programs, in the WNT signaling pathway [69–72], in DNA repair and cell senescence pathways, as reported for *NIPBL*-haploinsufficient cells [73]. Additionally, as recently reported, cells carrying pathogenetic variants in *NIPBL*, *SMC1A*, and *HDAC8* display spontaneous genome instability, increased oxidative stress, replicative senescence and accelerated cellular aging [74].

Focusing on impaired cohesin function and, specifically, on the fact that it may reduce DNA repair, Singh and colleagues advanced the hypothesis that persistent DNA damage could induce senescence and activate cytokine signaling in placental cells [75] (Fig. 2). They performed several experiments to test how cohesinopathy mutations impact DDR. Initially, DNA damage was assessed by analyzing placental-specific cell types in two different cohesin mutant backgrounds, trophoblast giant cells from *Nipbl*^+/−^ and *Hdac8*^−/0^ mice, finding that CdLS mice had elevated levels of histone H2AX phosphorylation, and thus DNA damage. To test whether DNA damage and DDR can actually worsen the senescence process, DNA damage was induced by γ-irradiation, and an ATM inhibitor was used to block the damage response. Hence, it was observed that ATM inhibitor significantly reduced SA-βgal activity, another well-known hallmark of senescence, in differentiated mouse trophoblast stem cells (TSCs), suggesting that this pathway could probably contribute to senescence process. Overall, the results of these experiments showed that in both TSCs and placental tissue an additional increase in DNA damage can aggravate the already existing process of senescence [75].

Furthermore, another publication showed that alterations in induced pluripotent stem cells derived from CdLS patients involved several markers of the senescence process: different cell morphology and motility, reduced proliferation, and abundance of p21 suggested a senescent phenotype. It has also been demonstrated that it is precisely cellular senescence that underlies many defects in mouse models of CdLS, suggesting that this could also be similar in humans and paving the way for possible therapeutic strategies for patients [76](Fig. 2).

Moreover, a correlation has emerged between the TGF-β pathway, already known to be involved in senescence process [77] and cohesinopathies such as CdLS. Remarkably, fibroblasts derived from patients with chronic atrial and intestinal dysrhythmia (CAID) (OMIM #616201) showed enhanced cell cycle progression, higher senescence, and higher activation of TGF-β signaling, indicating a clinical cross-talk between TGF-β and cohesin [78]. Despite the apparently conflicting mechanism, the phenomenon of geroconversion could occur. This process leads to a cell transition from quiescence to senescence. However, excessive growth stimulation might take place not only in growth-arrested cells but also in proliferating cells [78]. Indeed, the CAID syndrome is caused by a homozygous *SGO1* mutation (K23E), which encodes for the Shugoshin-1 protein that protects sister chromatid cohesion mainly to ensure chromosomal stability during mitosis and meiosis [79, 80].

### Rett syndrome

Rett syndrome (RTT, OMIM #312750) is a developmental neurological disorder caused by mutations affecting the methyl-CpG binding protein 2 (*MECP2*) gene, located on the X chromosome (Xq28), which is responsible for the disease in about 90% of classic RTT patients [81]. RTT almost exclusively affects females and, in males, causes severe neonatal encephalopathy, usually lethal in the first years of life [82]. The prevalence in the general population is about 1/25.000 and is estimated to be 1/10.000–15.000 live female births. Affected patients show normal development for the first 6–18 months of their life, which then arrests, and a period of regression occurs [83]. The main symptoms include intellectual disability, speech problems, hypotonia, and progressive motor dysfunction. Patients affected by RTT also show complications with breathing and heart rhythm defects, besides anxiety and social-behavioral problems [84].

The MeCP2 protein has a role in chromatin remodeling in both gene transcriptional activation and silencing; in particular, it interacts with histone deacetylases [85], histone methyltransferases BRM, SWI/SNF and ATRX [86, 87], but also with the DNA methyltransferase DNMT1, suggesting a possible role as a regulator and modulator of DNA methylation [88, 89]. MeCP2 protein is expressed in most tissues, although it is mainly found in the brain; in fact, MeCP2 protein has been shown to be central in neurogenesis and differentiation during early embryonic development, but more importantly, its expression is linked to neural maturation and synapse formation [90, 91].

Interestingly, Squillaro and colleagues demonstrated that the partial silencing of MECP2 in mesenchymal stem cells leads to a RTT-like phenotype but also induces a significant reduction of S-phase cells, along with an increase of G1 cells. These changes are accompanied by a reduction of apoptotic cells and an increase in cells undergoing the senescence process. In particular, senescence induced by reduced levels of MeCP2 is associated with DNA damage, activation of the Rb- and p53-related pathways, and an increase in SA-βgal expression [92, 93] (Fig. 2).

Among various hypotheses, it is supposed that Rett neurons do not show a lack of maturation but features of premature aging. Indeed, during the development of the disease, we assist to the induction of aging-related genes, including p53 targets [94]. Although there is a cognitive decline in young Rett patients and adults who exhibit several aspects related to the Parkinson’s phenotype, the typical phenotype does not exclusively present signs of premature aging [95, 96].

Reduced *MECP2* activity, therefore, induces decreased cell proliferation and triggers senescence in neural precursors. This process, in turn, could impair the formation and maturation of the neural cells themselves; it could disrupt decisions about neural cell fate and the maintenance of neurons [97]. Further studies are needed to understand whether the senescence process which cells undergo relates to the pathology, or whether the physiological loss of MeCP2 is indeed related to aging [96].

### Kleefstra syndrome

Kleefstra Syndrome (KLEFS, OMIM #610253) is a rare genetic neurodevelopmental disorder caused by point mutations in *EHMT1* (euchromatic histone-lysine N-methyltransferase 1) or, more frequently, by microdeletions of chromosomal region 9q34.3 (> 85% of cases), with loss of the entire gene [98]. This gene encodes for an enzyme, a histone methyltransferase that, methylating the lysine-9 position of histone H3 (H3K9me), modifies chromatin accessibility and is essential for normal development.

EHMT1 is particularly expressed in neuronal cells and plays an important role in maintaining homeostatic plasticity and synaptic remodeling; indeed, deletions in its coding gene induce changes in neuronal excitability and severe neurodevelopment [99].

The prevalence of KLEFS patients in the population is estimated to be around 1/200,000 individuals, and the diagnosis is based on characteristic clinical signs and molecular tests [100]. Cardinal phenotypic features include congenital generalized hypotonia resulting in delayed motor development, variable intellectual disability usually moderate to severe and associated with severe speech delay, autism spectrum behavioral disorders and distinctive facial features (i.e. some referable to the pathology with brachimicrocephaly, midfacial hypoplasia, abnormal eyebrow shape, synophria, Cupid’s bow upper lip and inverted lower lip). KLEFS patients at birth have anthropometric indices in the normal range; whereas, during adolescence more than 50% of individuals develop significant weight gain that can lead to overweight (BMI > 25) or obesity (BMI > 30) in adulthood [100–102].

Interestingly, a study of three adult patients with a defect in *EHMT1* outlined a phenotype exhibiting aspects of neurodegenerative progression (Fig. 2). Magnetic Resonance Imaging scans were conducted in the brains of these patients, and it was observed that behavioral and motor deficits became explicit after adolescence. Notably, the severity of these defects worsened over time, and multifocal subcortical signal abnormalities were found [103]. At the molecular level, the *EHMT1* gene appears to play also a role in aspects related to the neurodegenerative process. Indeed, repressive H3K9me2 was found to be significantly increased in the hippocampus of FAD mice (model of Alzheimer’s disease) (Fig. 2). By inhibiting *EHMT1* and *EHMT2*, which catalyze H3K9me2, the recovery of synaptic function and transmission lost during the onset of Alzheimer’s was observed, together with an improvement of cognitive deficits in aged FAD mice [103]. Therefore, it can be assumed that treating chromatin modifications regulated by euchromatin methyltransferases EHMT1/2 can effectively restore memory-related cognitive defects.

In addition, EHMT1 and EHMT2 methylate and silence the promoters of the *IL6* and *IL8* genes, restricting their transcription. Specifically, the degradation of these methyltransferases lifts this repression, leading to the upregulation of IL-6 and IL-8, key components of the SASP [104].

All these evidences further demonstrate that abnormal epigenetic mechanisms are central to the onset of neurodegeneration, to which senescence process might contribute [105].

## Senescence in rare lung diseases

Senescence in lung tissue has to consider the constant contact with external (e.g. pollutants, antigens, tobacco smoke) and host (microaspiration, gastroesophageal reflux, and commensal microbes) organic and inorganic stressors. In addition, the accumulation of senescent cells in lungs physiologically occurs with age together with a peculiar chronic, low-grade, systemic inflammatory state that accompanies the aging process (inflammaging). This contributes to establishing a senescent milieu in the pulmonary tissue.

The increased and accelerated cellular senescence has been identified as the basis for the onset and progression of pulmonary fibrosis and interstitial lung diseases (ILDs), a heterogeneous group of lung disorders characterized by inflammation and fibrosis of the lung parenchyma. Among ILDs, chronic obstructive pulmonary disease (COPD) is the fourth leading cause of death in developed countries (World Health Organization, 2021). It causes slow progressive airflow obstruction and emphysema due to the destruction of the lung parenchyma, a common feature of aging lungs. In both COPD patients and animal models, hallmarks of senescence and SASP establishment are present and have an impact on disease exacerbation [106]. Additionally, SASP paracrine signaling activates naïve cells in the microenvironment, induces tissue remodeling or alters intercellular interaction, eventually promoting pathogenetic mechanisms in the whole body. Indeed, the presence of telomere shortening in circulating cells and SASP factors in the plasma of COPD patients suggests the involvement of a systemic aging process in the development of non-lung manifestations and co-morbidities [107]. This might explain why atrophy and weakness, atherosclerosis and kidney dysfunction are associated with respiratory insufficiency.

**Fig. 3:**
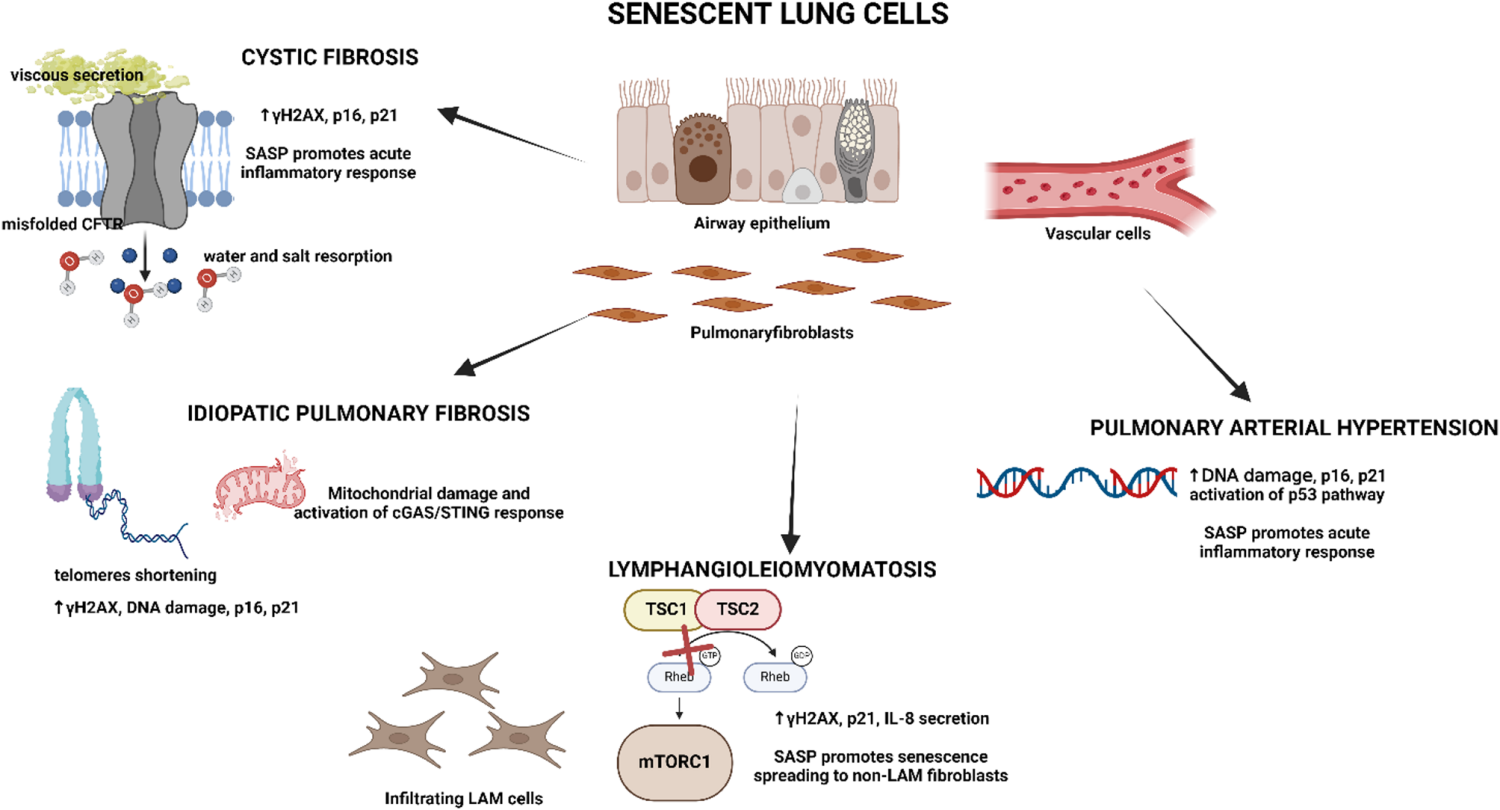
Senescence in rare lung diseases. Graphical representation of the senescence and SASP involvement in the described disorders. The central features and the major evidence are reported.

This paragraph will explore senescence involvement in pathological progression in four rare ILDs with similar characteristics to COPD. All these disorders are characterized by a basal state of inflammation and an elevated presence of SASP factors in both patient serum and lung tissues, indicating their important contribution to microenvironment remodeling and disease exacerbation (Fig. 3).

### Cystic fibrosis

Cystic Fibrosis (CF, OMIM #219700) is an autosomal recessive disease affecting 1/2500 newborns, with the highest incidence in the Caucasian population and Northern European descendants. The patients manifest persistent productive cough, hyperinflation of the lungs, and airway obstruction. Acute events often result in lung fibrosis and induce a permanent loss of lung function [108].

CF is caused by mutations in the cystic fibrosis transmembrane conductance regulator (CFTR) gene on chromosome 7, resulting in folding defects of the CFTR protein, an ATP-binding cassette transmembrane chloride channel located in the apical membranes of epithelial cells. Misfolded CFTR is dysfunctional and accumulates in lung epithelial cells after proteasome-mediated ubiquitination [109]. This results in the loss of intra/extra-cellular homeostasis, increased salt and water resorption, and the production of a thick and viscous secretion. In addition, recurrent infections cause the accumulation of inflammatory cells and the subsequent damage of bronchi, leading to the loss of cartilaginous support and muscle tone, and eventual bronchiectasis [108].

Chronic but ineffective airway inflammation is a substantial characteristic of CF, directly correlated with the genetic basis of the disease. The high presence of several cytokines and growth factors is observed in the bronchoalveolar lavage fluid (BALF) of both CF patients and in the CFTR-KO murine model [110, 111] (Table 1). They are involved in the activation of acute inflammatory response (IL-1α, IL-6, IL-8), stimulation of innate immunity (MCP1, IL-12), neutrophil migration (KC, murine orthologue of CXCL1) and survival (G-CSF). Interestingly, most of these pro-inflammatory soluble factors are components of the SASP, suggesting that the high basal state of inflammation also sustains senescence, which might be one of the mechanisms that contribute to CF exacerbation (Fig. 3). Indeed, the expression of senescent biomarkers such as p16, γH2A.X and p-Chk2 is high in goblet, ciliated and basal cells of CF epithelium compared to healthy controls [112] (Fig. 3).

One of the effects of stress-induced senescence is the increase of intracellular calcium concentration, a condition that, interestingly, is already constitutive in CF cells due to the genetic defects. Calcium accumulates in the mitochondria leading to their dysfunction following the loss of their membrane potential, the increased ROS production and NF-κB-dependent SASP release (IL-1α, -6, -8) [113].

Among SASP factors, VEGF levels are elevated in CF patients’ serum and BALF, potentially contributing to bronchiectasis-associated angiogenesis [114], negatively correlating with hypoxia, and being reduced by antibiotic therapy [115]. VEGF mRNA and protein expression are increased in CF lungs, and, in in vivo and in vitro CF models, high VEGF-A levels are related to CFTR dysfunction [116] (Table 1). At the state of the art, VEGF role in association with senescence in CF has not yet been explored, but its involvement in pathologic tissue alteration and disease progression suggests the need for further investigations.

### Idiopathic pulmonary fibrosis

Idiopathic Pulmonary Fibrosis (IPF, OMIM #178500) is a chronic, progressive, fibrosing interstitial pneumonia limited to the lungs and with unknown etiology. It usually occurs in patients older than 60 years who present reduced pulmonary function and/or impaired gas exchange. The prevalence is 0.3/4.51 cases per 100,000 individuals in the general worldwide population; the incidence and mortality are higher in men and increase with age [117].

The most recognized theory on IPF pathogenesis suggests that the repeated injury of alveolar epithelial cells (AECs) could lead to abnormal activation and impaired tissue repair, cytokines and growth factors release, which might trigger myofibroblast activation, ECM deposition, parenchymal destruction and lung function decline [118].

In the first years of this century, by investigating the basis of IPF, multiple mutations have been described in telomerase genes *TERT* (telomerase reverse transcriptase), which encodes for the catalytic telomerase reverse transcriptase, and *TERC* (telomerase RNA component), the RNA component used as a template. These mutations result in a reduction of telomerase activity from 60 to 10% of wild type protein. A significant shortening of telomere length in peripheral blood cells of IPF patients compared to age-matching controls results from the *TERT* and *TERC* mutations, but it is also observed in absence of any telomerase mutation [119, 120].

In IPF tissues, the identification of telomere-associated foci, a sign of unresolved telomere DNA damage, together with the increased expression and co-localization of γH2A.X and the high expression of p16 and p21 in remodeled areas are characteristic hallmarks of cellular senescence [121, 122] (Fig. 3). Additionally, p16 expression in IPF epithelial cells and fibroblasts correlates with disease severity and a decrease in lung function [121].

With a machine learning approach, Aversa and colleagues identified a subset of plasmatic senescence biomarkers and SASP factors that correlate with pulmonary function and accurately identify IPF patients: RAGE, MCP1, MMP-7, IL-10, GDF-15, α and VEGF-A [123]. Among these factors, the plasma levels of GDF-15, a stress-induced cytokine, component of SASP, is the most specific in identifying the presence of the disease.

Compared to healthy controls, IPF patient-derived fibroblasts present myofibroblast phenotype, lower replication rate, irregular shape and larger morphology, shorter telomeres and heightened mRNA and protein expression of p21 and p53. Moreover, the mRNA levels of the SASP factors IL-1β, IL-6 and FGFβ are increased [124]. (Table 1). In an in vitro co-culture model, senescent IPF-fibroblasts reduce proliferation and increase the migration of immortalized alveolar type II epithelial cells (A549) likely through their secreted factors, demonstrating the capability of senescent IPF cells to modify the microenvironment [125].

Senescence development in IPF fibroblasts is strictly related to mitochondrial dysfunction in an auto-feedback loop involving mTORC1 activation, and by ROS activity since the senescent phenotype is restored by antioxidant treatments [126]. IPF patient-derived lung fibroblasts release high amounts of sEVs (LF-EVs), which induce concentration-dependent epithelial cell senescence, mitochondrial dysfunction and ROS production. These effects are due to the IPF LF-EVs transfer of miR23b-3p and miR494-3p which act on SIRT3 in target cells [127]. Primary and secondary senescence in IPF may be driven by the release from AECs of high amount of mtDNA into cytosolic and extracellular spaces, that causes the activation of the cyclic guanine monophosphate-adenine monophosphate synthase and the induction of a type I IFN pro-inflammatory response via STING/NF-κB pathway activation, which increases SASP expression and release, and lung fibrosis [128] (Fig. 3). In in vitro and in vivo studies, senolytic treatment induces apoptosis in AECs, by reducing fibrosis and increasing epithelial cell function [129]. Among released factors, alveolar type II cells (ATII), progenitors of the alveolar epithelium, highly secrete PAI-1 (Plasminogen activator inhibitor 1 or SERPINE-1), which significantly contributes to the senescence establishment [130]. PAI-I has a role in fibrinogenesis, and it is involved in cellular adhesion, migration and proliferation [130] and negatively correlates with lung function in IPF patients [131]. PAI-1 inhibition almost completely blocks TGFβ1/p16-induced ATII senescence, the secretion of fibrinogenic mediators via p53/p21/Rb pathway and the macrophages activation [132].

### Pulmonary arterial hypertension

Pulmonary arterial hypertension (PAH, OMIM #178600) is a severe and progressive subtype of pulmonary hypertension (PH) affecting distal pulmonary arteries [133]. It can have idiopathic, heritable, virus or drug-induced origins or can occur as a complication of hypoxic lung diseases, including COPD and IPF. In the global population, PAH incidence is 3 to 5/100,000 middle-aged and older adults, with wide variability among the populations [133].

PAH is caused by the occlusive remodeling of the neointima of pulmonary vessels, small arteries thickening and muscularization of precapillary arterioles. ECM expansion progressively leads to vascular fibrosis, increased resistance to blood flow and pulmonary arterial pressure, vessel occlusion and right ventricular failure [134]. The vasculature remodeling originates from plexiform lesions, a hallmark of the PAH.

Van der Feen and colleagues hypothesized that the progression of degenerative changes in the pulmonary vasculature is caused by the establishment of a senescent status in the pulmonary epithelium and smooth muscle cells [135]. Initial cellular DNA damage in vessels induces a hyperproliferative state and oxidative stress, growth arrest and SASP establishment. Accumulation of senescent cells physically obstructs vessel lumen, leading to an irreversible sclerotic end-stage damage.

In PAH, Sirt1/PGC-1α (sirtuin1/peroxisome proliferator activated receptor gamma co-activator-1α) pathway inhibition in cardiac and pulmonary artery cells contributes to mitochondrial dysfunction and senescence [136]. Moreover, a global mitochondrial abnormality appears to contribute to the pathological involvement of remote organs (e.g. right ventricle) during PAH progression [137]. Mitochondrial alteration causes energy metabolism disorders, altered proliferation and ROS accumulation in human and rodent-derived PAH pulmonary arterial smooth muscle cells (PASMCs), increasing oxidative stress and inflammation. PARP-1 inhibition in PAH PASMCs restores mitochondrial membrane potential [138].

Hallmarks of senescent features are identified in patient tissues and different PH and PAH animal models obtained with monocrotaline (MCT)-treatment or by inducing hypoxia [139]. In pulmonary arterial endothelial cells (PAECs) of PAH patients the positivity to p16, p21, SA-βgal assay and γH2A.X is higher than in controls [140] (Fig. 3). Consistently, increased p21 and p16 protein expression has also been demonstrated in the lung of hypoxic or old mice [141] and in MCT-injured rats, together with the high positivity to MMP-2, IL-6 and TNFα, whose levels correlate with the disease severity [139] (Table 1). Interestingly, p16 knockdown and the senolytic treatment with ABT263 reduce epithelial to mesenchymal transition in PAECs of a hypoxia-induced murine model [140].

Integrated transcriptome analysis and machine learning algorithm identified five senescence biomarkers in peripheral blood of idiopathic PAH patients: TNF receptor superfamily member 1b, associated to cell differentiation, apoptosis and immune response; CCL-16, a chemotactic cytokine, angiogenesis and lymphangiogenesis stimulator; glutamate cysteine ligase modified units, involved in glutathione-dependent oxidative resistance and ferropoptosis-related factor; cytokine IL-15, a regulator of cell survival and related to immune response; superoxide dismutase 1 which protects from oxidative stress [142].

In PH and PAH lesions and serum, several angiogenic factors, including the SASP mediator VEGF and its receptors VEGFR-1 and − 2, have been found increased, suggesting their potential role as diagnostic and prognostic biomarkers. Interestingly, circulating levels of soluble VEGFR-1 are reduced after PAH-targeted treatments [143].

SASP establishment in PAH pathogenesis and exacerbation is also suggested by the increased blood levels of several pro-inflammatory cytokines, including TGFβ, MCP1, TNFα and different interleukins (e.g. IL-1α/β, -2, -6, -8), which expression is associated with patient survival [144–146] (Table 1). Indeed, the exposure of healthy PASMCs to TNFα and IL-6 induces DNA damage [138], and IL-6 overexpression in the PAH mouse model causes intimal thickening, pro-proliferative apoptotic resistant milieu, inflammatory cell recruitment and distal arterial muscularization [147]. The IL-6-induced damage can be limited by PARP-1 inhibition [138], which is involved in NF-κB-dependent transcription of pro-inflammatory cytokines.

Considering that PASMCs accumulation in the arterioles of the lung is an important characteristic of PAH, it is relevant to understand the mechanism underlying the cell recruitment and the driving factors. In this perspective, the platelet-derived EVs, an established tool of cell communication, in PAH patients’ serum is higher compared to healthy subjects [148]. In vitro experiments demonstrate that exosomes derived from murine senescent endothelial cells increase the PASMCs migration and accumulation in the mouse lung [140]. Moreover, plasma or lung tissue-derived EVs from MCT-injured mice lead to PH development in healthy mice and increased muscularization of pulmonary arteries [149]. Supporting the role of exosomes in PAH, PAH rat-derived EVs cargo are enriched of proteins and miRNA associated with PAH pathogenesis, including cellular respiration and vasculature remodeling (complement family, hemopexin, ceruloplasmin protein, miR-145, miR-451, miR-10b, miR-486-5p, mir-26a and miR-342) [148–150]. miR-486-5p and miR-26a-5p pro-angiogenic signaling is downstream regulated by NF-kB [148]. Moreover, the exposure to PAH model-derived EVs causes the in vitro and in vivo upregulation of IL-6, endothelin-1, erythropoietin receptor [149], ROS accumulation and induces the transcription and release of VEGF-A, FGF2 and VEGFR-1 and tube formation in PAECs [148]. In addition, the assessment of an oxidative cargo (high lipid peroxidation and NADPH oxidase activity) and low expression of PGC-1α and Sirt1 in circulating EVs of rat model indicate their functional role in the development of mitochondrial and oxidative stress changes in other districts (as heart and brain), and suggest their central role in the remodeling of remote tissues that occur in PAH [151].

### Lymphangioleiomyomatosis

Lymphangioleiomyomatosis (LAM, OMIM #606690) is a pulmonary low-grade, destructive, metastasizing neoplasm occurring almost exclusively in women, frequently during their childbearing age [152]. The global prevalence of LAM is estimated as 3.4/7.8 per million women although this might be an underestimation due to the disease rarity and patient variability and to the healthcare infrastructures in the different geographical areas [153]. LAM arises as sporadic lung disease or in 30–40% of adult females affected by the autosomal dominant genetic disease Tuberous Sclerosis Complex (TSC) (LAM/TSC).

Patients suffer from progressive dyspnea, chronic cough, fatigue, recurrent pneumothorax, chylothorax, and occasional hemoptysis associated with increased angiogenesis and lymphangiogenesis [152]. LAM lesions, as multifocal nodules and 2–5 mm cysts are constituted by a heterogeneous environment of normal lung cellular populations and LAM cells, smooth muscle-like cells characterized by loss of growth control, anaerobic glycolytic metabolism, high motility and invasiveness. These key features contribute to local and remote tissue invasion and destruction [152], as LAM patients often present extra-pulmonary manifestations, the most recurrent of which is renal angiomyolipomas [154].

In LAM cells, the loss of heterozygosity in *TSC1* or, more frequently, *TSC2*, in accordance with Knudson’s “two-hit model” or due to an epigenetic modification, drives the absence of hamartin or tuberin transcription, respectively [155, 156]. The genetic defect affects the formation of TSC complex, a key inhibitor of mTORC1, leading to its constitutive activation [157]. mTOR pathway constitutive activation is a major driver of senescent cell accumulation and metabolism alteration in COPD and pulmonary fibrosis, and it induces disease exacerbation [158]. The basis of LAM onset and progression are unknown, and cellular senescence might be one of the underlying mechanisms considering the role of mTORC1 hyperactivation in both LAM and senescence establishment.

The absence of Tsc1 or Tsc2 in murine embryo fibroblasts and PASMCs causes the reduction of in vitro growth potential and the insurgence of early onset of senescent features, as the p53-mediated p21 expression [158, 159] (Fig. 3). The pro-inflammatory SASP establishment following mTORC1 hyperactivation induces tissue remodeling, PH and emphysema in murine models [158, 160]. Senescent lung epithelium shows increased phosphorylation of mTORC1/PGC-1 suggesting the association with mitochondrial biogenesis and dysfunction [161]. Interestingly, aberrant mitochondria are also observed in patient-derived LAM cells [162].

In primary LAM/TSC cells isolated from chylothorax of a LAM/TSC patient [155], a mTORC1 hyperactivation-dependent senescent phenotype has been recently demonstrated by the high SA-βgal positivity, γH2A.X and p21 expression [163]. These LAM/TSC cells are characterized by a peculiar SASP, as they release high levels of the pro-inflammatory factors IL-1α, -6 and − 8, VEGF and MMPs involved in ECM remodeling [155, 163, 164] (Table 1). In an in vitro model that resembles the LAM microenvironment primary lung fibroblasts acquired senescent features when grown in the conditioned medium of primary LAM/TSC cells, demonstrated by positivity to senescent biomarkers, and increased the secretion of IL-8, suggesting the capability of LAM cells to spread senescence through SASP [163]. Furthermore, as for PH and emphysema, mTOR inhibition by rapamycin or induction of tuberin expression reduces senescent features of LAM cells [163].

Cytokine involvement in LAM is demonstrated at several levels. In LAM patient specimens, i.e. bronchoalveolar lavage fluid and lung tissue, chemokines MCP1 (also known as CCL2), CXCL1 and CXCL5, have been found at higher levels compared to age-matching donors [165]. Similarly, despite a certain degree of heterogeneity, high expression of chemokine receptors (CCR1, CCR2, CCR3, CCR7, CCR10, CXCR2, CXCR4, CXCR6 and CXC3R1) have been described in LAM lung tissue [165, 166]. Polymorphisms of MCP1, whose levels are directly associated with TSC2 deficiency, common in LAM patients, are associated with reduced lung function; moreover, LAM cells are selectively mobilized by MCP1, suggesting a paracrine feedback loop [165]. MCP1 is highly expressed in the BALF of a female murine LAM model, as well as several cytokines such as TNFα and TGFβ1, IL-1β, IL-6 and CXCL1 [167, 168] (Table 1).

VEGF-C and VEGF-D, SASP components and mitogen factors for vascular and lymphatic endothelial cells, are increased in LAM lesions likely controlling the lymphangiogenesis and lymphatic involvement in bronchioles, and, thus, the disease progression [168, 169]. VEGF-D level is high in the serum of LAM patients and correlates positively with lymphatic involvement and LAM severity [170, 171]. VEGF-D is a serum biomarker for the LAM diagnosis that, when above the threshold of 800 pg/mL, helps to distinguish LAM from other lung diseases with a specificity of 100% and a sensibility of 58% [170, 171]. Moreover, in Tsc2-mutated rat cells, Cui and colleagues showed the correlation between the mRNA levels of VEGF-D and MCP1 and their decrease after rapamycin treatment [172].

Among the SASP components implied in LAM, MMPs, ECM-degrading enzymes, drive parenchymal lesion formation and appear to facilitate the entrance of invasive cells in circulation. The expression of MMP-1, 2, 9, 11, 14 and 19 is increased in LAM lesions, and the MMP-2, 7 and 9 are upregulated in LAM cells and in the serum of LAM patients [173–175]. MMP-2, 3 and 9 are increased in the BALF of a LAM murine model and their levels decrease upon rapamycin and simvastatin combination therapy, together with lesion growth reduction [168]. Besides the MMPs contribution to neighboring and distant tissue invasion and the MMPs possible use as potential prognostic and therapeutical biomarkers, these data underline the key functional role of the SASP components as MMPs in LAM sustaining the role of senescence in the disease pathogenesis [164, 173–175].

**Table 1:** Increased SASP factors in lung diseases

Disease	Cytokines	Growth factors	Proteases
**CF**	IL-1α/β ^(111)^, IL-6 ^(111)^, IL-7 ^(111)^, IL-8 ^(110)^, IL-17 ^(111)^, CCL-3 ^(110)^, CCL-4 ^(110)^, CCL-20 ^(110),^ MCP1 (110), G-CSF ^(111)^, KC ^(111)^	VEGF ^(116)^	
**IPF**	IL1β ^(124)^, IL-6 ^(124)^, IL-7 ^(123)^, GDF-15 ^(123)^, MCP1 ^(123)^, PAI-1 ^(130)^	FGFβ ^(124)^	MMP-1 ^(123)^
**PAH**	IL-1 α /β ^(144)^, IL-6 ^(139,144,145)^, IL-8 ^(145)^, IL-13 ^(144)^, CCL-16 ^(142)^ TNFα ^(144)^, MCP1 ^(146)^	TGFβ ^(144)^	
**LAM**	IL-1α ^(155)^, IL-1β ^(168)^, IL-6 ^(155,167,168)^, IL-8 ^(155,163)^, CXCL1/KC (165,167,168), CXCL5 (165), MCP1 (165,167), TNFα (168)	TGFβ1 ^(167)^, VEGF-C ^(169)^, VEGF-D (168,170,171,173)	MMP-3 ^(167,168)^

SASP components (cytokines, growth factors and proteases) involved in the described lung disorders are shown

## Conclusions

Cellular senescence is a process triggered by different stressor stimuli, causing both epigenetic alterations and modifications of the surrounding microenvironment. This phenomenon has also been observed in rare disorders, which manifest with different phenotypes.

A well-established correlation between rare diseases and senescence is known for progeroid syndromes such as Hutchinson-Gilford Progeria syndrome (HGPS, OMIM #176670), Werner syndrome (WS, OMIM #277700) and Cockayne syndrome (CS, OMIM #216400, OMIM #133540), which are characterized by accelerated ageing [176] Interestingly, despite the monogenic nature of these disorders, they show molecular features of ageing similar to those seen during normal ageing. However, in certain rare disorders where aging features are subtler, the senescence process has been investigated to a lesser extent. Thus, here we describe two classes of rare diseases in which the main senescence features are depicted by epigenome alteration or SASP establishment, offering two types of models not only for the study of the senescence process, but also for investigating possible therapeutic approaches. Considering that, in principle, epigenome changes are reversible and the SASP detrimental effects can be suppressed by senomorphic drugs, deepening these studies could envisage new strategies for these rare disorders and improve translational research.

Chromatin remodeling is fundamental for the transcription of genes associated with cellular maintenance and cell fate; thus, it is essential to understand the epigenetic-based aberrations in the senescence process. The pivotal role of epigenetic landscape in senescence is well known and emerging studies have shown that epigenetic alterations and pioneer transcription factors, some of which are defective in MDEMs, can regulate SASP expression [177]. However, the mechanism of senescence in MDEMs has been so far poorly explored, and this review reported its involvement in four different chromatinopathies. Specifically, the transcription of specific genes that drive the process is central, whereas less relevant appears to be the involvement of the secretome. Thus, reviewing the senescence features reported in some MDEMs highlights the possibility to explore this process in chromatinopathies studies to elucidate its involvement as well as to offer a new perspective for pharmacological interventions, considering the increasingly growing field of senotherapies for a wide range of diseases [178].

On the other hand, in lung diseases, intercellular communication affects the entire microenvironment by establishing a pro-inflammatory milieu or inducing progressive and unrelenting parenchyma destruction The involvement of senescence in IPF was extensively demonstrated in in vitro models exploiting both fibroblasts and alveolar epithelial cells, indicating also a key function of SASP in its propagation. Similarly, senescence biomarkers were identified in the serum of PAH patients, as well as in tissues from patients and animal models. In addition, the senescent features of LAM/TSC cells and their capability to spread senescence to PLFs strengthens the hypothesis to consider senescence as a key player in LAM. However, further in vitro studies are required to demonstrate a strong correlation between senescence and CF exacerbation, given the different stimuli that might trigger the chronic inflammatory state that characterizes this disease. It is important to underline that secreted factors belonging to SASP might also be released also in conditions different from senescence, making necessary careful characterization studies. Therefore, a cellular senescent phenotype has to be clearly assessed before exploring the possible role of these soluble mediators in propagating senescence in the surrounding tissue and distal districts.

Thus, this review gives preliminary insights into state-of-the-art studies on the contribution of senescence to the pathogenesis of rare disorders, suggesting the need for further investigation. This could be fundamental not only for age-related diseases and cancer, but this subject should be considered to fill the current knowledge gap on pathogenetic mechanisms drivers of rare disorders, from which a personalized medicine perspective can benefit.

## Electronic supplementary material

Below is the link to the electronic supplementary material.

## Data Availability

Data sharing is not applicable to this article as no datasets were generated or analyzed during the current study.

## Abbreviations

AECs: Alveolar epithelial cells
ATII: Alveolar type II cells
ATM kinase: Ataxia-Telangiectasia Mutated kinase
BALF: Bronchoalveolar lavage fluid
BMI: Body mass index
CCL: C-C motif ligand
CCR: CC chemokine receptors
CDK: Cyclin dependent kinase
CdLS: Cornelia de Lange syndrome.
CF: Cystic fibrosis
CFTR: Cystic fibrosis transmembrane conductance regulator
COPD: Chronic obstructive pulmonary fibrosis
CXCL: C-X-C motif ligand
CXCR: CXC chemokine receptors
DDR: DNA damage response
ECM: Extracellular matrix
EHMT1: Euchromatic histone-lysine N-methyltransferase 1
EVs: Extracellular vesicles
G-CSF: Granulocyte colony stimulating factor
GDF-15: Growth/differentiation factor 15
HDAC: Histone deacetylase
IL: Interleukin
ILDs: Interstitial lung diseases
IPF: Idiopathic pulmonary fibrosis
KC: Keratinocyte chemoattractant, the murine orthologue of CXCL1
KLEFS: Kleefstra Syndrome
LAM: Lymphangioleiomyomatosis
LAM/TSC: Lymphangioleiomyomatosis associated to tuberous sclerosis complex
MCP1: Monocyte chemoattractant protein, also known as CCL2
MCT: Monocrotaline
MDEMs: Mendelian disorders of the epigenetic machinery
MeCP2: Methyl-CpG binding protein 2
MiDAS: Mitochondrial dysfunction-induced senescence
MMPs: Metalloproteases
mTOR: Mechanistic target of rapamycin
NF-κB: Nuclear factor of kappa light polypeptide gene enhancer in B-cells
NSD1: Nuclear receptor binding SET domain protein 1
PAECs: Pulmonary arterial epithelial cells
PAH: Pulmonary arterial hypertension
PAI-1: Plasminogen activator inhibitor 1, also known as SERPINE-1
PARP-1: Poly (ADP-ribose) polymerase-1
PASMCs: Pulmonary arterial smooth muscle cells
PH: Pulmonary hypertension
Rb: Retinoblastoma protein
ROS: Reactive oxygen species
RTT: Rett syndrome
SA-βgal: Senescence associated β-galactosidase
SASP: Senescence associated secretory phenotype
SIRT: Sirtuin
SMC1: Structural maintenance of chromosomes-1
SS: Sotos syndrome
TGF: Transformed growth factor
TNF: Tumor necrosis factor
TSC: Tuberous sclerosis complex
VEGF: Vascular endothelial growth factor
VEGFR: Vascular endothelial growth factor receptor
γH2A.X: Phosphorylated form of the histone variant H2A.X
